# Clinical Assessment of Fetal Well‐Being and Fetal Safety Indicators

**DOI:** 10.1002/jcph.2126

**Published:** 2022-09-15

**Authors:** Anna L. David, Rebecca N. Spencer

**Affiliations:** ^1^ Elizabeth Garrett Anderson Institute for Women's Health University College London London UK; ^2^ National Institute for Health and Care Research (NIHR) University College London Hospitals NHS Foundation Trust (UCLH) Biomedical Research Centre London UK; ^3^ School of Medicine University of Leeds Leeds UK

**Keywords:** adverse event, clinical trial, fetal therapy, fetus, pregnancy, safety

## Abstract

Delivering safe clinical trials of novel therapeutics is central to enable pregnant women and their babies to access medicines for better outcomes. This review describes clinical monitoring of fetal well‐being and safety. Current pregnancy surveillance includes regular antenatal checks of blood pressure and urine for signs of gestational hypertension. Fetal and placental development is assessed routinely using the first‐trimester “dating” and mid‐trimester “anomaly” ultrasound scans, but the detection of fetal anomalies can continue throughout pregnancy using targeted sonography or magnetic resonance imaging (MRI). Serial sonography can be used to assess fetal size, well‐being, and placental function. Carefully defined reproducible imaging parameters, such as the head circumference (HC), abdominal circumference (AC), and femur length (FL), are combined to calculate an estimate of the fetal weight. Doppler analysis of maternal uterine blood flow predicts placental insufficiency, which is associated with poor fetal growth. Fetal doppler analysis can indicate circulatory decompensation and fetal hypoxia, requiring delivery to be expedited. Novel ways to assess fetal well‐being and placental function using MRI, computerized cardiotocography (CTG), serum circulating fetoplacental proteins, and mRNA may improve the assessment of the safety and efficacy of maternal and fetal interventions. Progress has been made in how to define and grade clinical trial safety in pregnant women, the fetus, and neonate. A new system for improved safety monitoring for clinical trials in pregnancy, Maternal and Fetal Adverse Event Terminology (MFAET), describes 12 maternal and 18 fetal adverse event (AE) definitions and severity grading criteria developed through an international modified Delphi consensus process. This fills a vital gap in maternal and fetal translational medicine research.

Conducting clinical trials in pregnancy raises many challenges, primarily associated with safety concerns for the mother and the fetus, and particularly when testing novel maternal and fetal therapies. The legacy of thalidomide and diethylstilbestrol teratogenicity has meant that traditionally, pregnant women have been excluded from participating in clinical trials of therapeutics. They are considered to be a “vulnerable” population in research because of their developing fetus.^1^ Pregnancy is often a wonderful time for most women, but for many expectant mothers it is a complex journey associated with anxiety, uncertainty, and fear because of concerns about their health and that of their baby. Increasingly, our maternal populations have health issues before they conceive, often in association with obesity and older maternal age. Optimizing their pregnancies using effective pharmaceuticals is challenging. Ideally, this should begin before conception, but many women conceive without being able to adequately prepare for pregnancy. There is currently no legal or regulatory requirement for new drugs to be tested on pregnant women. Thus >80% of pregnant patients routinely receive therapies that have not been adequately studied in pregnancy.^2^ Clinicians and patients are often unaware of this evidence gap about the drugs that they are prescribing or ingesting.^3^ The resulting underinvestment and inequality in women's health and the health of their unborn children leaves them exposed to a major health inequality, as many drugs used in pregnancy undergo only post‐marketing surveillance.

The initial routine exclusion of pregnant women from COVID‐19 clinical trials is a particular case in point.^4^ For many trials, exclusion was not well justified as the treatments being evaluated had no or low safety concerns during pregnancy. In common with many virus infections in pregnancy, multiple observational cohort studies showed that pregnant women have higher rates of severe COVID‐19 infection than non‐pregnant women, and more pregnancy complications such as preterm birth and pre‐eclampsia.^5^

But perhaps the more pressing problem is the millions of women and children who die each year during pregnancy and childbirth from obstetric complications such as preterm birth, fetal growth restriction (FGR), pre‐eclampsia, and hemorrhage. Globally, preterm birth is the second leading cause of childhood death <5 years of age, affecting 1 in 10 infants in the United States. These common conditions alone make it a moral imperative to include pregnant women in therapeutic trials.^6^ Yet the Concept Foundation has recently highlighted that since the 1990s, there have only been 2 drugs developed and registered for pregnancy‐specific conditions.^7^

Governments, regulators, researchers, women and their families, and the pharmaceutical industry are engaging to identify methodologies to generate data to better inform medicine use in pregnancy. Initiatives such as the Task Force on Research Specific to Pregnant Women and Lactating Women (PRGLAC) have focused on gaps in knowledge and research on safe and effective therapies for pregnant or lactating women and the fetus.^8^ Other initiatives include ConcePTION, developed by the Innovative Medicines Initiative (IMI), which has established a system to generate reliable evidence‐based information on pharmaceutical use for pregnant and lactating women.^9^ A major limitation when considering fetal pharmacology and therapeutics is assessing clinical safety and efficacy, as the fetus sits in an environment that is in many ways highly protected but is extremely vulnerable to interventions. This review describes ways in which maternal and fetal well‐being can be examined, all of which will allow the assessment of the effect of therapeutics in pregnancy. This ensures that data on maternal and fetal well‐being are accurately collected using currently available techniques. There are innovations becoming available in maternal, fetal, and placental monitoring, and progress has been made in how to assess clinical trial safety in pregnant women, the fetus, and neonate.

## Clinical Assessment of the Fetus and the Placenta

Assessing the impact of an intervention on a pregnant trial participant involves both the mother and the fetus. The placenta tells the story of the whole pregnancy, from its early development and anatomical configuration, through its maturation and tolerance of labor and delivery, and therefore provides a useful summary of the fetal journey. Examining the direct effect of an intervention on the fetus and the placenta before birth is more challenging. However, there are promising developments in fetal heartbeat and movement monitoring, fetal and placental imaging, and markers for fetal and placental well‐being in maternal blood that may improve safety and efficacy monitoring in the future. Deep learning strategies are now being applied to ultrasound and magnetic resonance imaging (MRI) of the fetus and the placenta, providing new assessment methods through techniques including classification, segmentation, object detection, and tracking.

### The Placenta and the Fetal Membranes

The placenta has a wide range of functions. Commonly it is considered to act as the lungs and the gut for the developing fetus, transporting oxygen and nutrients from the mother. It is also responsible for the manufacture of proteins such as human placental lactogen (hPL) and human placental growth hormone (hPGH), in a similar way to the liver.^10^ These proteins act in concert to modulate maternal metabolism to meet the energy requirements of the developing fetus and can be detected in the maternal circulation, potentially providing a snapshot of placental function. The placenta also clears fetal waste products, acting as the kidneys for the pregnancy, that are excreted by the mother's kidneys. Maternal urine markers have been investigated to predict fetal well‐being, for example low urinary estriol, produced by the placenta and endocrine system of the fetus, is associated with small for gestational age (SGA) fetuses. These markers, however, have insufficient predictive power to be useful clinically.^11^ The placenta plays a key role in averting fetal infection, preventing many bacteria and virus particles from crossing to the fetus. The lack of placental expression of the 2 receptors for severe acute respiratory syndrome coronavirus 2 (SARS‐CoV‐2), ACE2 and TMPRSS2, which have been identified as a prerequisite for infection, means that the developing fetus is relatively well protected from COVID‐19 infection.^12^ This has been borne out by evidence from observational studies of COVID‐19 infection in pregnant women. Stem villi in the placenta play an important role as connective tissue to anchor and support the placenta in place within the uterus. The placenta also contains multipotent stem cells that can be differentiated into a variety of cell types.^13^ Finally, the placenta is important for maintaining fluid balance both in the fetus and in the amniotic sac, where it controls the passage of fluid across the chorionic plate, the amniotic and chorionic membranes, and the umbilical cord.

The whole placenta and membranes can be analyzed after birth, yielding useful information on the presence of infection (chorioamnionitis), fetomaternal hemorrhage such as placental abruption, and other pathologies such as maternal or fetal vascular malperfusion that are associated with placental insufficiency. Routine application of the Amsterdam consensus criteria to sample and histologically analyze the placenta facilitates the international comparability of clinicopathologic studies of the placenta in clinical trials.^14^

Amniotic fluid can also be collected for analysis at the time of cesarean section birth. The myometrium must be carefully incised down to the level of the amniotic membrane, after which a sterile quill or blunt needle can be introduced into the amniotic cavity and fluid drawn up into a sterile syringe. With experience, usually around 5 to 50 mL of sterile fluid can be easily collected.

Umbilical cord blood can be easily collected after birth, even when delayed cord clamping is performed to provide the fetus with a placental transfusion. Analysis of base excess, lactate, and umbilical artery or umbilical vein pH are used to indicate the degree of acute and/or chronic fetal acidosis prior to birth. Fetal hemoglobin concentration and hematocrit are also often measured by blood gas analyzers available on the delivery unit and can indicate fetal anemia or erythrocytosis.

Finally, the placental “bed”, the area of the placental attachment to the uterine decidua, can be biopsied under direct observation at cesarean section using a blade, biopsy forceps, or suction catheter, or after vaginal delivery under ultrasound guidance using transvaginal biopsy forceps.^15^, ^16^

In all cases, for optimal results the time from delivery to sample collection and processing should be kept short, preferably <30 minutes.^16^

### Sampling of Fetal and Placental Tissues Before Birth

Before birth, the collection of chorionic villi or amniotic fluid during pregnancy is feasible using ultrasound‐guided minimally invasive sampling. The technique is commonly used in fetal medicine for the prenatal diagnosis of fetal genetic abnormalities, such as aneuploidy and single‐gene disorders, in the context of a high‐risk aneuploidy screening test, abnormal ultrasound findings, or a significant family history. The amniotic fluid can also be collected to confirm diagnosis in the case of suspected congenital fetal infection such as cytomegalovirus, for example, which is excreted in fetal urine. Opportunistic amniotic fluid analysis is possible, for example, to aid in the monitoring of drug transfer from the mother into the amniotic fluid, if a pregnant woman taking a medicine decides to undergo amniocentesis for clinical reasons. Amniocentesis is recommended to be performed after 15^+0^ weeks of gestation and chorionic villus sampling (CVS) is advised from 11 weeks of gestation; both can be performed up to term. Amniocentesis can provide around 20 mL of fluid for analysis and CVS will yield around 5 g of chorionic villi, which will need to be cleared of any maternal cell contamination under a microscope. The additional risk of miscarriage following amniocentesis or CVS when it is performed by an appropriately trained operator is considered to be below 0.5%.^17^

Fetal blood can also be sampled via ultrasound‐guided cordocentesis, either at the site of the umbilical vein insertion into the placental cord insertion or as it traverses the fetal liver (intrahepatic umbilical vein). The net fetal loss rate as a result of cordocentesis is around 1% to 2%.^18^ International guidelines recommend sampling after 18 weeks of gestation as the risk of fetal loss is higher if performed in earlier gestations; sampling from the free cord loop is also associated with a higher risk of complications and should be avoided.^19^ In the 1980s, cordocentesis was used to demonstrate the severity of fetal hypoxia in small growth‐restricted fetuses and to correlate it with other markers of fetal compromise, such as hypercapnia, acidosis, and hypoglycemia.^20^ This provided definitive evidence that asphyxia was not solely associated with the process of birth but was commonly caused by placental insufficiency. Fetal blood sampling has also been used to confirm engraftment following in utero stem cell transplantation. Here, a male fetus with X‐linked severe combined immunodeficiency received an intraperitoneal stem cell injection of first‐trimester nucleated fetal liver cells at 14 weeks of gestation. At 10 and 19 weeks after transplantation, mixed chimerism was confirmed using genomic human leukocyte antigen (HLA) class‐II typing and flow cytometry in fetal blood collected by cordocentesis.^21^

Fetal blood can be accessed via ultrasound‐guided intracardiac injection, although this is performed in fetal medicine units far less often now than in the 1980s and 1990s because of the better safety profile of umbilical vein sampling. Intracardiac blood sampling tended to be used in the first trimester for prenatal diagnosis prior to the advent of rapid quantitative fluorescence polymerase chain reaction (QF‐PCR) analyses from amniocentesis and CVS samples. Other uncommon fetal tissue sampling techniques include ultrasound‐guided transabdominal liver biopsy for the analysis of enzyme activity to diagnose some congenital metabolic syndromes; the procedure has been mostly superseded by DNA analysis.

### Fetal Heart Rate Monitoring

Cardiotocography (CTG) or a non‐stress test is the external electronic detection of the fetal heart rate (FHR) and uterine activity via maternal abdominal monitors. This technique has been used clinically to assess fetal well‐being, either antenatally or intrapartum, since the 1970s. One concern with the use of CTG monitoring for fetal well‐being in clinical trials is its interpretation in preterm gestations of <32 weeks of gestation; this is even more of a difficulty in the context of congenital disease.^22^ The physiological control of the FHR differs in preterm and term fetuses. Some characteristics and patterns of the FHR are dependent on gestational age, as they reflect the development and maturity of the central nervous system as well as the cardiovascular system. Certain FHR features may be pathological in a term fetus but could instead be physiological in a preterm fetus; an example is reduced FHR variability, which may be a normal feature. In addition, there is an increased possibility of signal loss and poor‐quality CTG in the preterm fetus. Preterm CTG monitoring assessment has undergone detailed review in the National Institute of Healthcare Research (NICE) Preterm Labor and Birth guideline.^23^ The NICE committee agreed that guidelines for term fetuses could be considered as relevant for the fetus beyond 32 weeks of gestation, as the physiological maturity of the cardiovascular and neurological systems from this gestational age is comparable with that of the term fetus. Thus, at >32 weeks of gestation, baseline FHR and variability should be similar to that in the term fetus, and accelerations with an amplitude of >15 beats from the baseline should be present as an indicator of fetal well‐being. Similarly, decelerations can be interpreted as showing evidence of fetal compromise, as for the term fetus.

The interpretation of CTG at gestational ages of <32 weeks is more challenging, particularly <26 weeks of gestation when the fetal autonomic nervous system is immature. Generally, however, it can be agreed that a combination of abnormal CTG signs <32 weeks of gestation, namely a baseline FHR of >160 beats per minute (bpm), variability reduced to <5 bpm, and deep or prolonged decelerations, indicate a compromised fetus.^23^ At these extreme preterm gestational ages, CTG monitoring plans will need to be individualized to consider the likelihood of a good neonatal outcome were the fetus to be delivered immediately based on fetal concerns. Discussion with the parents ahead of fetal monitoring is recommended, so as to allow sufficient time to consider and agree a plan for early delivery, if that is decided upon.

Computerized analysis of the antenatal CTG (cCTG) is increasingly being used via the application of objective Dawes–Redman criteria, which assesses various features of the CTG trace according to several evidence‐based criteria. The standard features of visual assessment, such as accelerations, decelerations, and basal FHR, are included as well as parameters that are difficult or impossible to measure visually, such as short‐term variability (STV), sinusoidal rhythm, and the number of minutes of high variation.^24^ cCTG is commonly used to monitor antenatal fetal well‐being in clinical practice, particularly in the setting of high‐risk pregnancies such as those with SGA or FGR.^25^ For example, cCTG was 1 of the 3 fetal monitoring arms of the TRUFFLE clinical trial that investigated the optimum decision tool for delivery in the presence of early‐onset FGR.^26^ STV, which is assessed by cCTG, may more reliably detect fetal hypoxia compared with the traditional direct clinical visualization of CTG.

Fetal electrocardiogram (ECG) monitoring has also been applied in labor to detect intrapartum fetal hypoxia with limited success.^27^ Ongoing work using machine learning is being applied to both the intrapartum CTG and the intrapartum ECG, which may lead to a more reliable assessment of fetal well‐being in the future.^28^

### Fetal Movements and Fetal Breathing Movements

Other indicators of fetal well‐being include the presence of normal fetal body and breathing movements. These were previously assessed as part of the biophysical profile, a relatively labor‐intensive procedure involving up to 30 minutes of ultrasound observation of fetal movements and fetal breathing movements, fetal tone, fetal heart rate, and amniotic fluid volume.^29^, ^30^ As an alternative, fetal MRI has recently been used to visualize fetal movements, characterizing the mechanical stress and strain experienced by the developing human skeleton in utero.^31^ Advances in machine learning are also leading to the automation of fetal movement assessments and the development of wearables to allow longer‐term monitoring.^32^, ^33^ Such technology could improve the assessment of the short‐ and medium‐term fetal response to interventions.

Novel techniques to evaluate fetal neurodevelopment using 4‐dimensional ultrasound assessment of fetal movement may provide an early signal of fetal neurological deficits and fetal pain, an important consideration in trials involving interventions directly into the fetus.^34^, ^35^ Fetal pain, and maternal distress associated with it, was an important consideration for a Patient Public Advisory Group who took part in a Delphi consensus process to develop maternal and fetal adverse event (AE) terminology (discussed later).^36^ Procedures for fetal analgesia and anesthesia for interventions are highlighted by the recently updated Society for Maternal Fetal Medicine guidelines for fetal surgery.^37^

### Assessing Fetal Size, Structure, and Circulation Using Ultrasound

The assessment of fetal size, structure, and fetoplacental circulation using ultrasound is the mainstay of antenatal care in high‐resource settings. Increasingly it is being applied in low‐ and middle‐income countries (LMICs), with the advent of less expensive smaller portable scanners and even handheld devices that can connect to mobile phones. Ultrasound is used in the first trimester to confirm gestational age, using the measurement of the crown–rump length (CRL) up to 13^+6^ weeks of gestation, and the head circumference (HC) from 14 to 20 weeks of gestation. Verifying gestational age is an important part of antenatal care as it will determine the timing of potential decisions about the induction of labor and delivery towards the end of gestation, as well as define whether a pregnancy delivers preterm (<37 weeks of gestation). In the latter part of pregnancy, measuring fetal abdominal circumference (AC) and HC will allow an assessment of the size and growth of the fetus and will assist in the diagnosis and management of FGR, where the fetus fails to achieve its growth potential. This may be caused by several factors, most commonly placental insufficiency and maternal medical disorders, with genetic conditions (aneuploidy and single‐gene disorders), fetal structural anomalies, and infection being less common. The combination of AC, HC, biparietal diameter (BPD), and femur length (FL) in an equation, such as Hadlock's formula, for example, provides a more accurate estimate of fetal weight (EFW) than any of the parameters alone.^38^ A normal fetal growth trajectory is considered to indicate a healthy maternal–fetoplacental unit, although it is always considered in retrospect and does not necessarily indicate continuing fetal or maternal health. Growth patterns are helpful in distinguishing between different types of FGR, such as symmetrical FGR, which typically has an early onset of <32 weeks of gestation.^39^ Asymmetry between measures of head (BPD and HC) and abdominal (AC) size can be identified in asymmetrical FGR, which typically has a later onset (>32 weeks of gestation), and results in a brain‐sparing effect with a relatively larger head compared with the AC.

**Figure 1:**
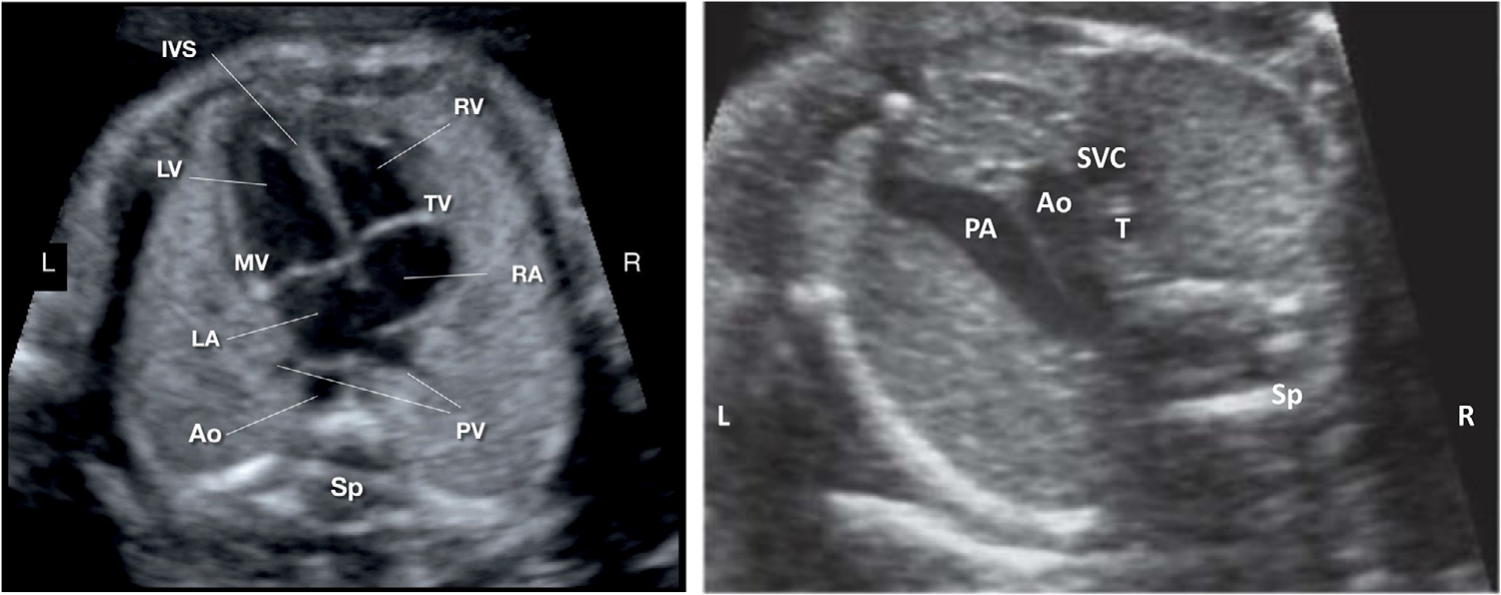
Ultrasound images of the fetal heart views used to screen for cardiac anomalies: the left‐hand image is of the 4‐chamber view and the right‐hand image is of the 3‐vessel and trachea view. Ao, aorta; IVS, interventricular septum; LA, left atrium; LV, left ventricle; MV, mitral valve; PV, pulmonary veins; RA, right atrium; RV, right ventricle; Sp, spine; SVC, superior vena cava; T, trachea; TV, tricuspid valve.

**Figure 2:**
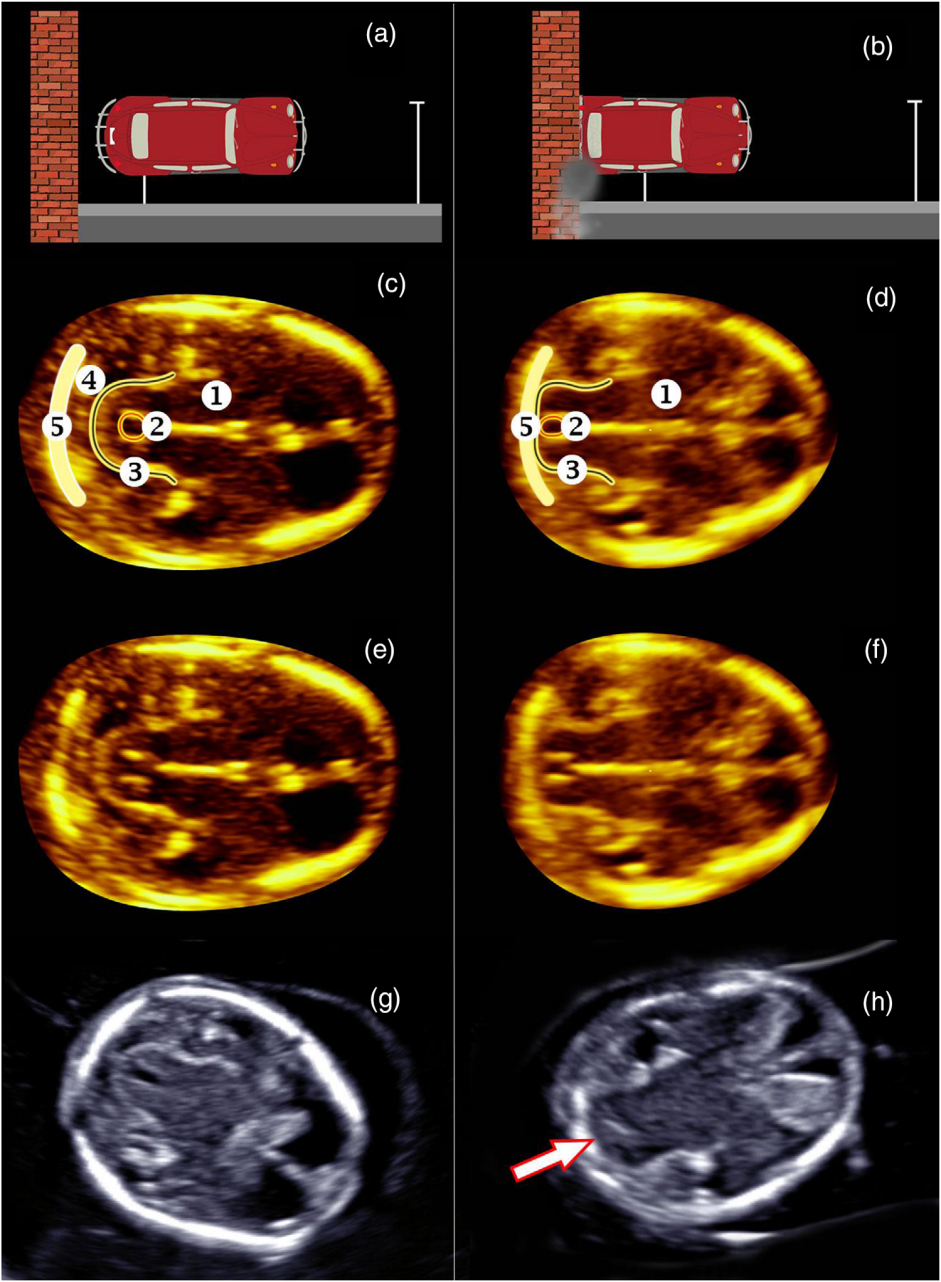
Crash sign: a first‐trimester sonographic marker of spina bifida. Schematic diagrams showing a car stationed away from a wall, representing the mesencephalon (car) and occipital bone (wall) in normal fetuses (a), and then reversed into the wall, representing the posterior displacement of the mesencephalon and deformation against the occipital bone (“crash sign”) in fetuses with open spina bifida (b). (c–h) Ultrasound images in axial view at 12 to 13 weeks of gestation, showing mesencephalon in normal fetuses (c, e, g) and the crash sign in fetuses with open spina bifida (d, f, h). (c–f) Three‐dimensional reconstructed images of 2 sets of monochorionic twins discordant for spina bifida. (g, h) Images of singleton fetuses without (g) and with (h) the crash sign (arrow). 1, thalamus; 2, aqueduct; 3, mesencephalon; 4, arachnoid space; and 5, occipital bone. Reproduced with permission from *Ultrasound Obstet Gynecol*. 2019;54(6):740–745 (first published: 11 April 2019); DOI: 10.1002/uog.20285.

Advances in the systematic assessment of sonographic fetal imaging planes has improved the detection rates of common fetal structural anomalies, such as cardiac defects. This is important for the detection of potential teratogenic drug effects. In the UK, the National Health Service (NHS) Fetal Anomaly Screening Programme (FASP) is using data from the National Congenital Anomaly and Rare Diseases Registration Service (NCARDRS) to demonstrate improvements in the detection of 4 major cardiac conditions (transposition of the great arteries, atrioventricular septal defect, tetralogy of Fallot, and hypoplastic left heart syndrome) following the application of specific fetal imaging views: the 4‐chamber view and the 3‐vessel and trachea view (Figure 1). Increasingly, fetal anomalies are being diagnosed in the first trimester following improvements in ultrasound technology such as 3D imaging. At the routine dating scan conducted at 11 to 14 weeks of gestation, for example, a finding of posterior displacement of the mesencephalon and deformation against the occipital bone called the “crash sign” is thought to occur as a result of reduced intracranial pressure, and can indicate a high chance of fetal myelomeningocele (open spina bifida) (Figure 2).^40^

**Figure 3:**
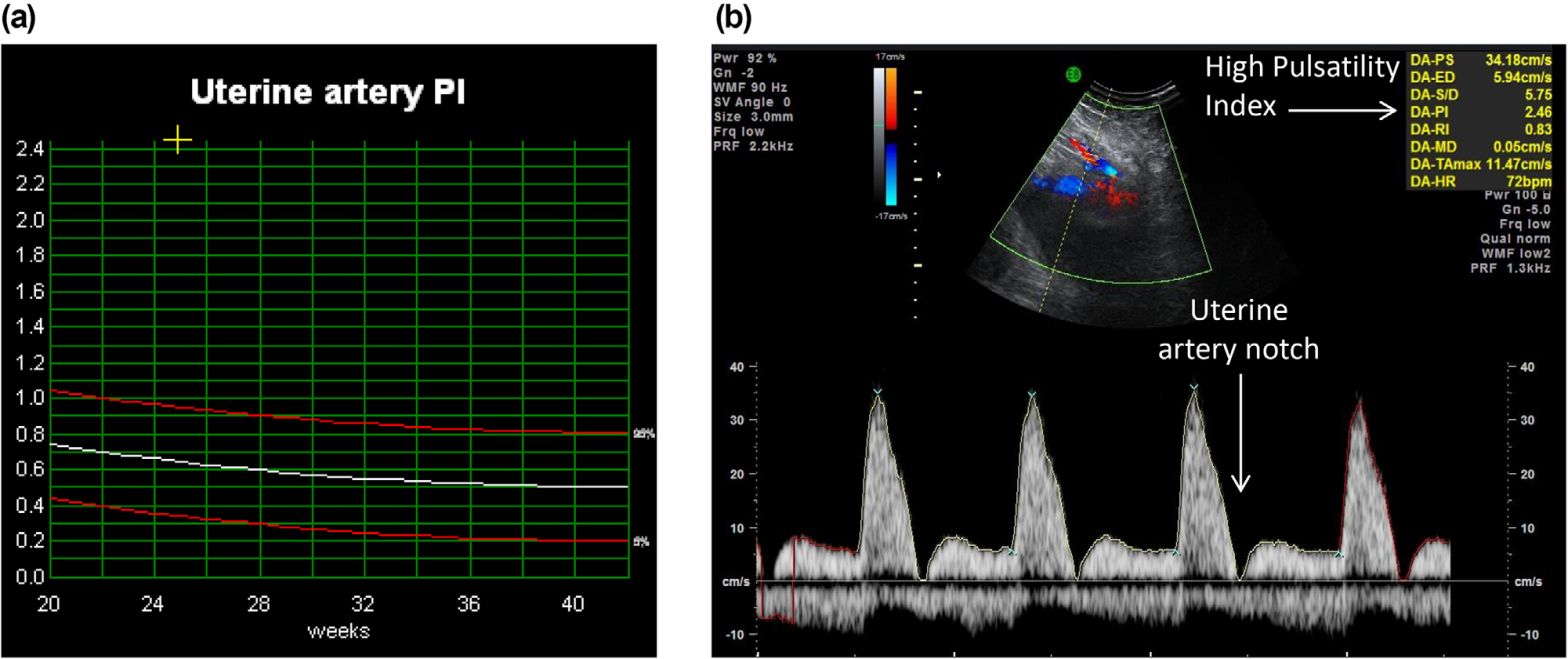
(a) Uterine artery waveform with diastolic notch. (b) Pulsatility index (PI) above the 97th centile, indicating high‐resistance circulation with placental insufficiency.

Doppler ultrasound allows the assessment of the velocity of blood within fetal and placental vessels and provides an indirect assessment of fetal and placental condition. Doppler studies of the uterine artery during the first and early second trimester may be used to predict pregnancies at risk of adverse outcome, particularly early‐onset pre‐eclampsia and FGR. Both conditions manifest via placental insufficiency, where the development of the uteroplacental circulation is reduced through the inadequate trophoblast transformation of the spiral arteries into high‐flow, low‐resistance vessels.^15^ Markers of increased resistance to uterine artery blood flow, including the diastolic “notch” in the waveform in early diastole, are thought to result from increased vascular resistance in the uteroplacental vascular bed and a raised pulsatility index (PI) (Figure 3). If identified in the first trimester, raised uterine artery PI can be used as part of a multifactorial screening assessment using maternal serum markers and mean arterial pressure to accurately predict the risk of early‐onset pre‐eclampsia.^41^ In a multicenter study of singleton pregnancies at 11 to 13 weeks of gestation, screening detected 100% (95%CI 80% to 100%) and 75% (95%CI 62% to 85%) of pre‐eclampsia < 32 and <37 weeks of gestation, respectively, at a 10.0% false positive rate. Consequently, such pregnancies can be closely monitored with increased surveillance for the possible development of maternal hypertension and proteinuria, and with serial assessment of fetal size.

Further ultrasound techniques are in development to measure placental volume and vascularity for the prediction of pre‐eclampsia and FGR. Two‐dimensional ultrasound measurements of first‐trimester placentation are correlated with fetal size but are not related to subsequent excessive or slow fetal growth.^42^ Fully automatic placental volume estimation can now be achieved in the first‐trimester placenta in real time using a validated computerized tool: OxNNet. In addition, a novel 3D image‐processing technique allows the rapid calculation of fractional moving blood volume (FMBV) to facilitate standardized measurement of the vasculature of the entire uteroplacental interface in the first trimester as a means to detect hypovascular placentas.^43^

### Magnetic Resonance Imaging

Magnetic resonance imaging (MRI) provides superior soft‐tissue definition compared with ultrasound, and overcomes several technical limitations of ultrasound, such as atypical fetal position, reduced amniotic fluid volume, or high maternal body mass index. Increasingly it is being used to complement ultrasound analyses, to improve the diagnosis of fetal anomalies following ultrasound detection, and/or for assessment prior to fetal surgery such as spina bifida closure.^44^, ^45^

Traditionally MRI has been used as a clinical adjunct to assess structural fetal anomalies in the central nervous system, but more recently it has been applied to other organ systems.^46^, ^47^ Fetal movements can cause artifacts in MRI, but various approaches can be used to reduce their negative effect on images, such as the adjustment of acquisition parameters. Super‐resolution reconstruction (SRR) is another tool that combines images captured in the 3 orthogonal planes to generate 1 volume for analysis of the fetal brain and neck masses.^48^, ^49^ Similar application of motion‐corrected slice‐to‐volume registration software has been shown to improve the diagnosis of fetal cardiac lesions when compared with 2D MRI.^50^ MRI volumetry can provide precise estimates of total lung volume, which may have a potential role in predicting the outcome of congenital diaphragmatic hernia, congenital lung lesions, and potentially neonatal lung function.^51^

Another promising area is the MRI measurement of placental function, including oxygenation within the maternal and fetal placental compartments, and fetal circulation. MRI is safe in pregnancy and the whole placenta may be imaged at any gestational age.^52^, ^53^ Dynamic contrast‐enhanced magnetic resonance imaging (DCE‐MRI) is a technique that enables the spatial and quantitative characterization of the maternal perfusion of the placenta, where fast imaging sequences, for example gradient echo, are repeatedly applied over the organ of interest during bolus administration of a contrast agent.^54^ As contrast agents such as gadolinium, for example, may cross to the fetus and be recirculated in the amniotic fluid, their use is only recommended clinically if it significantly enhances diagnostic performance and is expected to improve fetal or maternal outcome, as in the case of placental attachment spectrum (PAS) disorders such as placenta accreta.^55^ Non‐contrast agent‐based techniques such as diffusion imaging are consequently being keenly developed to measure placental function, providing information on diffusion within placental tissue and the exchange properties of maternal and fetal circulations. These include arterial spin‐labeled (ASL) MRI, which takes advantage of the magnetic pre‐labeling of blood as it passes into the field of view, and diffusion‐weighted (DW) MRI, which uses the random motion of water molecules within tissue as contrast.^56^ Oxygen can also be used as a surrogate contrast agent through maternal inhalation via blood oxygen level‐dependent (BOLD) MRI, where changes in placental oxygen saturation can be measured, giving an indication of placental dysfunction.^57^

**Figure 4:**
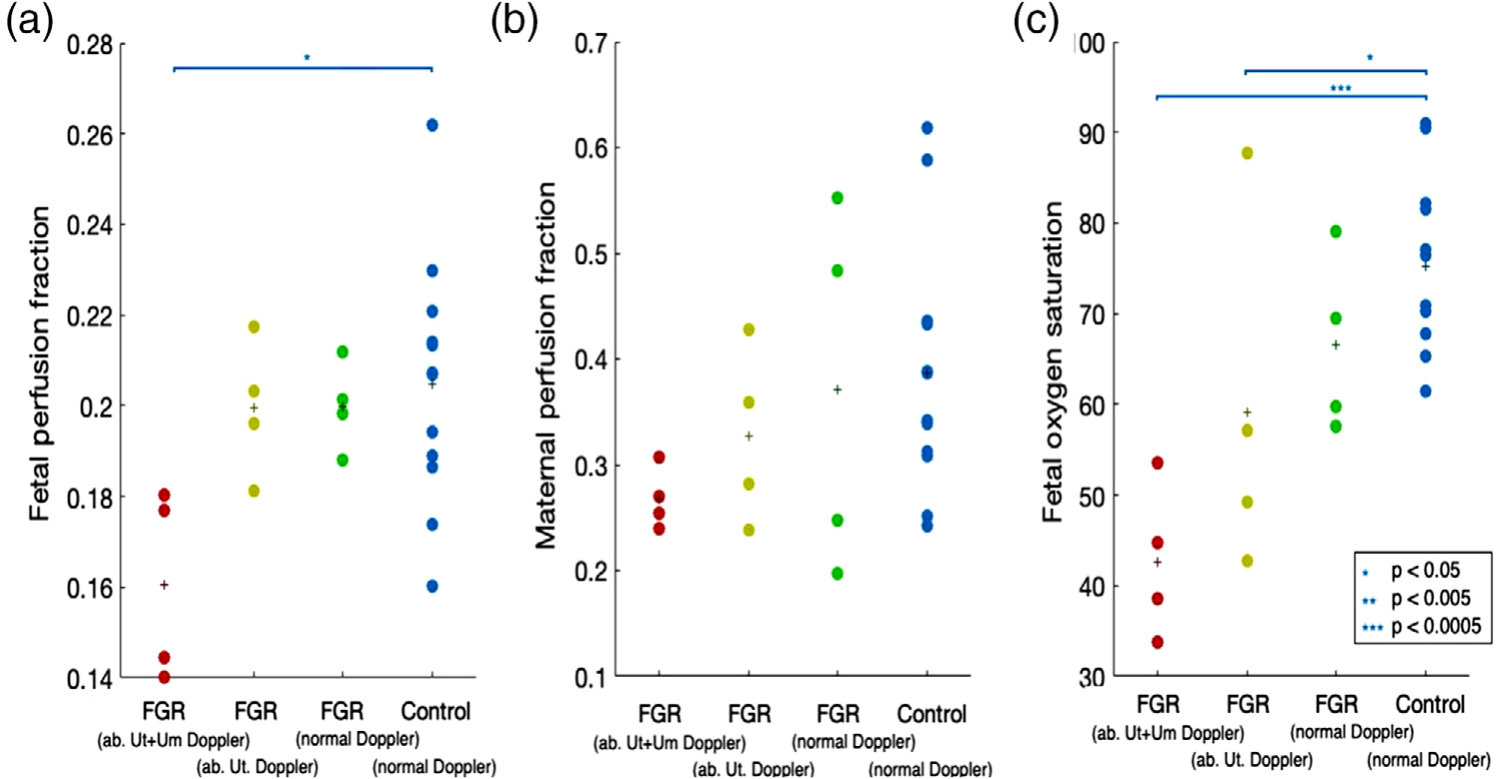
MRI‐derived maternal and fetal perfusion fraction and fetoplacental blood oxygen saturation with gestational age using the DECIDE multimodal algorithm in early‐onset fetal growth restriction (FGR) and control age‐matched normal pregnancies. Fetal and maternal perfusion and fetal oxygen saturation were determined in women grouped according to severity of early‐onset FGR. Key: red dots, FGR with uterine and umbilical artery Doppler >95th centile (abnormal uterine and umbilical Doppler FGR, n = 4); yellow dots, FGR with uterine artery Doppler >95th centile and umbilical artery Doppler <95th centile (abnormal uterine Doppler FGR, n = 4); green dots, FGR with umbilical and uterine Doppler <95th centile (normal uterine and umbilical Doppler FGR, n = 4); blue dots, control (n = 12). There are significant differences in the fetal perfusion fraction between the groups (0.16 ± 0.02 versus 0.20 ± 0.02 versus 0.20 ± 0.01 versus 0.20 ± 0.03, *P* = .048, groups as described above) with post hoc analysis showing that the difference lay between the abnormal uterine and umbilical Doppler FGR group (0.16 ± 0.02) and the control group (0.20 ± 0.03). There was also a significant difference in MRI‐derived fetoplacental blood oxygen saturation (42 + 7 ± 8.5 versus 59.2 ± 20.0 versus 66.5 ± 9.9 versus 75 ± 9.6%, *P* = .0079, groups as described above), with a significant difference between the abnormal uterine and umbilical Doppler FGR group and the normal Doppler FGR group (*P* = .006) and the control group (*P* = .0005). Group mean values shown as plus signs. *P < .05, **P < .005; ***P < .0005. Reproduced with permission from *BJOG* 2020;128(2):337–345 (first published: 30 June 2020); DOI: 10.1111/1471‐0528.16387. ab, abnormal; FGR, fetal growth restriction; MRI, magnetic resonance imaging; Um, umbilical; Ut, uterine.

Diffusion‐weighted (DW) MRI has been combined with intravoxel incoherent motion (IVIM), a measure of the microscopic translation of water molecules within a voxel during MRI, to provide an algorithm assessment of placental diffusion and perfusion changes, called DECIDE. This technique has been used to demonstrate reduced fetoplacental oxygenation in pregnancies affected by early‐onset FGR compared with gestational age‐matched control normal pregnancies (Figure 4).^58^ Moreover, DECIDE identified that compared with the left lateral position, maternal supine position in healthy late pregnancy is associated with reduced uteroplacental blood flow and oxygen transfer across the placenta. There was an average 6.2% reduction in oxygen delivery to the fetus and an average 11% reduction in fetal umbilical venous blood flow.^59^ This may be a possible mechanism behind the known association between supine sleeping and late pregnancy stillbirth. The findings demonstrate the huge potential that this computational MRI technology holds for the future understanding of placental function, an important consideration when testing drugs in pregnancy. Such measurements would be especially important as indicators of efficacy in trials of therapies for pre‐eclampsia, FGR, and preterm labor, and they could also provide safety signals in trials of therapeutics that have the potential to affect uterine perfusion.

### Monitoring Using Maternal Circulating Proteins

Non‐invasive prenatal diagnosis of fetal aneuploidy, single‐gene disorders, and blood group through the analysis of circulating DNA in maternal blood samples is well established in clinical practice. Placentally produced proteins can provide surrogate markers of placental function and/or damage, such as lower maternal serum concentrations of placental growth factor (PlGF) in placental insufficiency and pre‐eclampsia.^60^ PlGF is now being used clinically to confirm the diagnosis of pre‐eclampsia and to predict the likelihood for delivery within 2 weeks.^61^ It may also have utility in the management of pregnancies with SGA (EFW < 10th centile).^62^

The study of circulating messenger RNA (mRNA) and microRNA (miRNA) markers may allow for a more detailed assessment of placental gene expression and function while providing non‐invasive indicators of fetal hypoxia.^63^ Factors such as maternal body mass index and the maternal contribution to products that are not placenta specific are limitations of measuring circulating proteins and RNA in maternal blood. A more targeted assessment of the placenta may be possible by analyzing the cargo of placental extracellular vesicles, lipid‐bound structures containing proteins, and RNA from their tissue of origin, which could act as a “liquid biopsy” of the placenta.^64^

## Better Communication About Safety: Maternal and Fetal Adverse Event (AE) Consensus

Conducting clinical trials in pregnancy faces many challenges, primarily with regards to safety concerns for the mother and fetus, particularly when testing novel maternal and fetal therapies. The paucity of clinical trials in pregnancy has led to absent standard frameworks such as standardized severity grading for maternal and fetal AEs. This renders clinical trials in pregnancy more difficult and can compromise the health of pregnant participants.

Although they may not necessarily have a causal relationship with the investigational drug, AEs are important signals in clinical trials, facilitating the swift and responsible communication of safety data between study investigators, sponsors, and regulators.^65^, ^66^ AEs should be recorded in medical records and reported to the sponsor and other relevant authorities. A decision should then be made as to whether they meet the regulatory definition of “serious” and are directly related to the administration of the investigational drug. This will determine whether to classify the event as a serious adverse reaction (SAR). AE severity is recorded using standard grading criteria, commonly the Common Terminology Criteria for Adverse Events (CTCAE) (v5.0), which comprises 837 potential AEs.^67^ The grading of AEs allows decisions around dose escalation to be taken more objectively and also permits comparison of AEs between clinical trials. The CTCAE contains AEs related to “pregnancy, the puerperium, and perinatal conditions”, including fetal death and/or growth retardation, premature delivery, pregnancy, puerperium, and other postnatal conditions. Some condition‐specific severity grading for pregnancy‐specific events have been developed (eg, HIV‐AIDS and surgery).^68^, ^69^ However, until recently there were no standard general severity grading criteria. This contrasts with Delphi consensus work to integrate neonatal terminology and definitions into wider dictionaries, undertaken by the International Neonatal Consortium.^70^, ^71^ The Neonatal Adverse Events Severity Scale v1.0 classifies neonatal AEs into 5 grades (mild, moderate, severe, life threatening, or death), with severity defined by the effect of the AE on age‐appropriate behavior, basal physiologic functions, and healthcare changes in response to the AE.

Through an international Delphi consensus process involving healthcare professionals and patient groups, a team has systematically developed definitions and severity grading for maternal and fetal AEs, called MFAET.^36^ Fetal AEs had to be diagnosed in utero, with the potential of severe AEs to cause a detrimental effect before birth. New fetal AE definitions were developed by considering the different organ systems that might be affected and were aligned with the Medical Dictionary for Regulatory Activities (MedDRA) in liaison with their Maintenance and Support Services Organization (MSSO) in 2016.^72^ A generic fetal grading system was based on CTCAE criteria (Table 1) and then AE severity was graded independently for the pregnant woman and for the fetus (Table 2). Two example fetal AE definitions, fetal fluid collection and fetal cardiac function abnormalities, are illustrated in Table 1. These 12 new maternal and 18 fetal AE definitions and severity grading criteria were then ratified by consensus and realigned with MedDRA in 2022 to create MFAET v1.1. The terminology fills a vital gap in maternal and fetal translational medicine research and supports the development of therapies for pregnant women and their neonates.^43^

**Table 1:** General Principles of MFAET Grading and 2 Examples of Fetal AE Definitions and Grades

Fetal AE	Grade 1 (Mild)	Grade 2 (Moderate)	Grade 3 (Severe)	Grade 4 (Life‐Threatening)	Grade 5 (Death)
*Generic grading*	Clinical observation of uncertain significance. Resolves spontaneously. Low risk of long‐term consequences.	Likely to resolve spontaneously. Low risk of long‐term consequences. Requires increased frequency of monitoring, but less than once a week. Requires additional tests.	Requires increased frequency of monitoring, of once a week or more. Likely to lead to significant neonatal morbidity.	Likely to lead to fetal injury or permanent disability. Likely to lead to neonatal death. Requiring a substantive change in management, including changing the course of an interventional procedure or necessitating delivery.	Fetal death
*Fetal fluid collection* Definition: The collection of non‐hemorrhagic fluid in one or more fetal compartments (pericardial space, pleural space, peritoneal cavity, skin)	–	New‐onset isolated pericardial, pleural, or peritoneal fluid collection or skin edema that is not life‐threatening.	New‐onset accumulation of fluid in at least 2 fetal compartments (hydrops) that resolves spontaneously.	New‐onset accumulation of fluid in at least 2 fetal compartments (hydrops) that is sustained. Life‐threatening isolated pericardial, pleural, or peritoneal fluid collection.	Fetal death
*Fetal cardiac function abnormalities* Definition: An abnormality in fetal cardiac function	–	–	Non‐life‐threatening signs of cardiac failure, including cardiomegaly and valve regurgitation.	Likely to lead to fetal injury or permanent disability. Requiring a substantive change in management, including changing the course of an interventional procedure or necessitating delivery.	Fetal death

AE, adverse event; MFAET, Maternal and Fetal Adverse Event Terminology.

The severity of the AE is graded independently for the pregnant woman and for the fetus, as pregnancy conditions can affect the mother and the fetus separately. Fetal AEs are defined as being diagnosable in utero with the potential to cause detriment to the fetus.^36^

**Table 2:** MFAET, the Maternal and Fetal Adverse Event Terms for Which Definitions and Severity Grading Criteria Were Developed

Maternal AEs	Fetal AEs
Hemorrhage in pregnancy	Hemorrhage in pregnancy
Preterm premature rupture of membranes	Preterm premature rupture of membranes
Chorioamnionitis	Chorioamnionitis
Anemia in pregnancy	Anemia in pregnancy
Gestational hypertension	Fetal fluid collection
Pre‐eclampsia	Fetal bradycardia: non‐labor
Eclampsia	Fetal tachyarrhythmia
Premature labor	Cardiac function abnormalities
Puerperal infection	Fetal brain scan abnormal
Postpartum hemorrhage (primary)	Fetal gastrointestinal tract imaging abnormal
Retained placenta or membranes	Fetal musculoskeletal imaging abnormal
Amniotic fluid embolism	Fetal renal imaging abnormal
	Fetal movement disorders
	Fetal neoplasm
	Fetal structural abnormalities: not otherwise classified
	Abnormal fetal growth
	Fetal intraoperative injury
	Procedural hemorrhage
	Post‐procedural hemorrhage

AE, adverse event; MFAET, Maternal and Fetal Adverse Event Terminology.

The system has now been aligned with MedDRA v1.1, February 2022.^36^

## Conclusion

Assessing fetal and maternal well‐being using existing clinically relevant techniques is feasible, but novel technologies will allow us to monitor placental function and fetal oxygenation more closely. Even before the COVID‐19 pandemic shone a light on the inequities of drug development for pregnant and lactating persons, there was investment from agencies both public and private to address this issue. There is new guidance to facilitate scientific and ethical considerations for including pregnant and lactating persons in clinical trials, as well as new safety terminology to define and grade AEs in the mother, fetus, and neonate. Progress is now being made in concert with patients and the public to overcome the barriers to drug development and prescribing in pregnancy and lactation.

## Conflicts of Interest

A.L.D. receives consulting fees from Esperare Foundation, Geneva, Switzerland, a private not‐for‐profit organization, as chair of the Data Safety Monitoring Board in a clinical trial of an investigational fetal drug therapy. She is an unpaid co‐chair of the Maternal Health Project Group of the Association of British Pharmaceutical Industry (ABPI). She is a commissioner (unpaid) on the University of Birmingham's Policy Commission on Safe, Effective and Accessible Medicines for Use in Pregnancy. R.N.S. has no financial interests to disclose.

## Funding

The research leading to these results has received funding from the European Union Seventh Framework Programme (FP7/2007‐2013) under grant agreement no. 305823 (EVERREST), the EGA Hospital Charity, and through an Innovative Engineering for Health award (GIFT‐Surg) by the Wellcome Trust (WT101957) and Engineering and Physical Sciences Research Council (EPSRC) (NS/A000027/1). A.L.D. is funded at the National Institute for Health Research (NIHR) University College London Hospitals Biomedical Research Centre. R.N.S. is funded by the NIHR as a Clinical Lecturer.

## Author Contributions

A.L.D. substantially contributed to the conception or design of the work, drafted the work, and revised it critically for important intellectual content. She approved the version to be published and agrees to be accountable for all aspects of the work. R.N.S. contributed to the design of the work and drafting the article. She provided approval of the version to be published and agrees to be accountable for all aspects of the work.

## Data Sharing

All data and information are publicly available through the references.
